# Provider-patient communication about Zika during prenatal visits

**DOI:** 10.1016/j.pmedr.2017.05.003

**Published:** 2017-05-18

**Authors:** Fangjian Guo, Alexander R. Norton, Erika L. Fuchs, Jacqueline M. Hirth, Mariano A. Garcia-Blanco, Abbey B. Berenson

**Affiliations:** a Department of Obstetrics & Gynecology, The University of Texas Medical Branch, Galveston, TX, United States; b Center for Interdisciplinary Research in Women’s Health, The University of Texas Medical Branch, Galveston, TX, United States; c Department of Biochemistry and Molecular Biology, The University of Texas Medical Branch, Galveston, TX, United States; d Programme in Emerging Infectious Diseases, Duke Medical School, Singapore

**Keywords:** Pregnancy, Zika virus, Provider-patient communication, Prenatal, Travel, Mosquito

## Abstract

Zika virus transmission within and between the Americas is of global concern. This study assessed knowledge about the Zika virus among pregnant women in the United States, their travel plans to endemic areas, and whether their health care providers discussed Zika with them.

This cross-sectional study used data from 492 pregnant women (18–50 years) from an online survey conducted from April 8 to July 27, 2016. Pregnant women were recruited online through Facebook, Twitter, Craigslist, and Reddit.

Almost all (97.8%) participants had heard of the Zika virus, of which 71% first learned about it from the internet. Over one third of these pregnant women reported that their health providers discussed transmission of the Zika virus with them. Most respondents reported that their providers had discussed risks related to travelling to areas with Zika outbreaks. Half of the survey respondents reported that their providers gave them information about avoiding mosquito bites. Pregnant women were not concerned about Zika affecting their own health, but 34% were very or extremely concerned about it affecting their babies' health. Almost no pregnant women currently had travel plans to areas with ongoing Zika transmissions, and of the 14% who previously had plans, most (85%) cancelled their travel due to concerns about Zika.

Overall, pregnant women in our sample were highly knowledgeable about Zika virus. Over one third of women received suggestions regarding prevention of Zika from their healthcare providers.

## Highlights

• Almost all pregnant women in our sample are highly knowledgeable about Zika virus.
• One third of women receive information about Zika from their healthcare providers.
• Pregnant women are not concerned about Zika affecting their own health.
• Over one third of pregnant women are very or extremely concerned about Zika virus affecting their babies' health.
• Almost no pregnant women currently had travel plans to areas with ongoing Zika transmissions.

## 1 Introduction

Zika transmission in the Americas is of immediate global concern (Faria et al., 2016). Zika virus has already reached Puerto Rico and the southern United States (US) as well as the US Virgin Islands. As *Aedes* mosquitos are present in over half of the states in the US, there is substantial risk for introduction of Zika virus into the other parts of the US. Other vulnerable parts of the US for transmission include Hawaii, and additional states along the Gulf Coast, such as Texas. The virus is predominantly transmitted by infected *Aedes aegypti* mosquito, which bites primarily during the day but also at dusk and dawn. Blood transfusion and sexual intercourse are also possible routes for transmission (Foy et al., 2011, Oster et al., 2016).

Most (80%) Zika virus infections are subclinical and asymptomatic. In those who develop symptoms, patients typically present with fever, rash, conjunctivitis, headache and joint pain (Chen and Hamer, 2016). Those symptoms are generally mild and can resolve in a week. The viremia lasts about 1 week, but the duration of persistence of Zika virus in semen may be much longer with cases reported several weeks after resolution of Zika symptoms (Oster et al., 2016). Zika virus infection can cause Guillain-Barré syndrome among infected people (Cao-Lormeau et al., 2016), and microcephaly (Heymann et al., 2016, Mlakar et al., 2016, Cauchemez et al., 2016, Rasmussen et al., 2016) and other neurological defects (Martines et al., 2016, Calvet et al., 2016) in infants born to mothers infected during pregnancy. In February 2016, the World Health Organization (WHO) declared that recently reported clusters of microcephaly and other neurological disorders are a Public Health Emergency of International Concern (PHEIC) (Heymann et al., 2016). The Centers for Disease Control and Prevention (CDC) issued travel alerts suggesting pregnant women postpone travel to areas with ongoing Zika virus transmission, due to the association between Zika and birth defects. The CDC, the American College of Obstetricians and Gynecologists, and the Society for Maternal-Fetal Medicine have issued practical guidelines for health providers to help control Zika virus infection in pregnant women (Petersen et al., 2016, American College of Obstetricians and Gynecologists and Socie), including counseling about preventive measures. Whether these travel alerts and guidelines are being heeded by pregnant women and their health providers is unknown. This study aimed to assess characteristics associated with knowledge about Zika among pregnant women (18–50 years) in the US and whether their health providers had talked with them about Zika.

## 2 Methods

We conducted an online survey of pregnant women (18–50 years) from April 8 to July 27, 2016. Pregnant women were recruited via ads for participants with survey links on websites, including Facebook, Twitter, Craigslist, and Reddit. Typical text for the ads was ‘Pregnant? Please take this online survey and help Zika researchers.’ We administered the online survey via SurveyMonkey. Survey questions were adapted from the National Health Interview Survey (National Center for Health Statistics, n.d.), the First Nations' Knowledge of and Protection from the West Nile virus survey (First Nations Centre, National Aboriginal Health Organization, n.d.), and the Ipsos poll on Zika virus conducted for Reuters (Ipsos Public Affairs, n.d.). The survey included questions about participants' demographics, knowledge of Zika infection, their travel plans, and whether their health care providers had talked with them about Zika. The questionnaire was available in both English and Spanish (see online Supplemental material for detail). We received 580 responses in English and 5 in Spanish. Of those 585 respondents, 492 were available for analysis after excluding women who refused to provide consent (n = 3), < 18 years old (n = 16), not currently living in the US (n = 38), not currently pregnant (n = 29), and incomplete responses (n = 7).

Participants provided informed consent on the first screen of the online survey by responding to the following question, “Do you agree to the above terms? By selecting “Yes” and clicking the “Next” button, you are indicating that you are at least 18 years old, have read and understood this consent form, and agree to participate in this research study.” This study was approved by the Institutional Review Board at the University of Texas Medical Branch, Galveston, Texas.

## 3 Statistical analysis

Statistical analyses were conducted using SAS software version 9.4(SAS Institute; Carey, NC). A 2-sided p value < 0.05 was considered statistically significant. Descriptive analyses included chi-squared and Fisher's exact (when applicable) tests for categorical variables (e.g., race) and *t*-tests for continuous variables (e.g., age). Multivariate logistic regression models were used to assess factors associated with binomial outcomes, such as knowledge about Zika, receiving counseling about Zika from their health providers, and whether very or extremely concerned about Zika affecting their health. Variables that were controlled for included age, region of residence, country of birth, race/ethnicity, education level, and relationship status. Respondents with missing data were excluded from the analysis. As most of the respondents were recruited from Reddit, sensitivity analyses were performed by restricting analyses in those pregnant women.

## 4 Results

We charted the number of respondents from each state in the US on a US map (Supplemental Fig. 1). Among this national sample of 492 pregnant women, the mean age was 30 years and the median gestational age was 20 weeks. Most had a 4-year college degree or above, 93.1% were born in the US, and 86.6% were married or living with a partner (Supplemental Table 1). Almost all (97.8%) participants had heard of Zika.

Among these pregnant women, over one third reported that their health care providers had talked with them about Zika (Table 1). There were no demographic differences between women who had received information about Zika from their health care providers and those who had not (p values for chi-squared or Fisher's exact tests all > 0.05). Mean ages were also similar between those two groups (30.6 vs. 30.0, p = 0.13). Most (86%) of the respondents who had received information about Zika from providers reported that their providers discussed risks related to travel to areas with current Zika outbreaks, of which 94.8% of their providers suggested that they avoid travel to these areas (Supplemental Table 2). About half of the respondents who discussed Zika with a provider received information about avoidance of mosquito bites from their providers.

**Table 1:** Receiving information about Zika from health care providers among pregnant women (N = 492).

	n (%)	Proportion (95% CI)	Adjusted OR^a^
All	492 (100)	33.4 (29.2–37.6)	
Age			
≤ 30 years	261 (53.1)	32.9 (27.1–38.8)	Reference
> 30 years	231 (47)	33.9 (27.7–40.1)	1.02 (0.69–1.5)
Region of residence^b^			
South	216 (43.9)	35.1 (28.6–41.5)	Reference
Northeast	90 (18.3)	34.8 (24.9–44.8)	0.95 (0.56–1.6)
Midwest	76 (15.5)	34.2 (23.3–45.2)	0.95 (0.54–1.67)
West	110 (22.4)	28.3 (19.7–36.9)	0.73 (0.44–1.22)
Country of birth			
US	458 (93.1)	33.4 (29–37.8)	1.04 (0.48–2.24)
Other	34 (6.9)	33.3 (17.2–49.5)	Reference
Race/ethnicity			
Non-Hispanic White	421 (85.6)	34.1 (29.5–38.6)	1.16 (0.64–2.11)
Other	71 (14.4)	29.2 (18.1–40.3)	Reference
Education level			
Master's or doctoral degree	190 (38.6)	34.2 (27.4–41)	1.18 (0.68–2.06)
4-year college degree	189 (38.4)	35.3 (28.4–42.3)	1.27 (0.74–2.17)
No college degree	113 (23)	28.6 (19.9–37.2)	Reference
Relationship status			
Married	426 (86.6)	34.5 (30–39.1)	1.37 (0.71–2.63)
Other	66 (13.4)	25.8 (14.9–36.7)	Reference

a Adjusted odds ratio for receiving information about Zika from health care providers. It was adjusted for age, region of residence, country of birth, race/ethnicity, education level, and relationship status.

b Regions of residence were divided according to the following US Census Regions: South included Alabama, Kentucky, Mississippi, Tennessee, Arkansas, Louisiana, Oklahoma, Texas, Delaware, District of Columbia, Florida, Puerto Rico, Georgia, Maryland, North Carolina, South Carolina, Virginia and West Virginia; Northeast included Connecticut, Maine, Massachusetts, New Hampshire, Rhode Island, Vermont, New Jersey, New York and Pennsylvania; Midwest included Illinois, Indiana, Michigan, Ohio, Wisconsin, Iowa, Kansas, Minnesota, Missouri, Nebraska, North Dakota and South Dakota; West included Arizona, Colorado, Idaho, Montana, Nevada, New Mexico, Utah, Wyoming, Alaska, California, Hawaii, Oregon and Washington.

Among pregnant women who had heard of Zika, 71.1% first heard about this virus from the Internet and 19.3% from TV news. Most (90.4%) were aware of CDC recommendations for pregnant women regarding travel to areas with Zika outbreaks. Almost all (99.2%) pregnant women knew that Zika virus could be transmitted by mosquito bites, 90.9% knew that Zika virus could be transmitted by sexual contact, and only 2.3% incorrectly identified airborne transmission (Table 2). However, 79 (16.4%) incorrectly thought that there was local spread of Zika virus via mosquito bites in the continental US. This was before cases of Zika virus infection by local mosquitoes in Florida and Texas were reported. Most recognized that there is no cure for Zika infection, and 83.0% correctly identified fever as a common symptom. Almost all participants were aware of the association between Zika and birth defects, and were able to correctly identify microcephaly as one related birth defect. For knowledge about Zika (local transmission, route, symptoms, cure, and birth defect), 29.8% answered ≥ 1 of these 4 items incorrectly. Education level was the only sociodemographic factor associated (negatively) with incorrect knowledge.

**Table 2:** Knowledge about Zika virus among pregnant women aware of Zika (n = 473).

	n (%)		
	Correct	Incorrect^a^	Uncertain^b^
Route of transmission (n = 473)			
Sexual contact with an infected human?	430 (90.9)	14 (3)	29 (6.1)
Through sharing air with an infected person, especially if they are coughing?	357 (75.5)	11 (2.3)	105 (22.2)
The bite of an infected mosquito?	469 (99.2)	2 (0.4)	2 (0.4)
Symptoms (n = 471)			
Fever	391 (83.0)	2 (0.4)	78 (16.6)
Rash	314 (66.7)	11 (2.3)	146 (31.0)
Joint pain	279 (59.2)	6 (1.3)	186 (39.5)
Conjunctivitis	166 (35.2)	39 (8.3)	266 (56.5)
Muscle pain	248 (52.7)	9 (1.9)	214 (45.4)
Headache	269 (57.1)	9 (1.9)	193 (41.0)
Cure for Zika infection (n = 469)	367 (78.3)	5 (1.1)	97 (20.7)
Birth defect (n = 469)^c^	458 (97.7)	4 (0.9)	7 (1.5)
Microcephaly (n = 457)^d^	445 (97.4)	4 (0.9)	8 (1.8)

a Women who incorrectly answered the question. Women who answered “Don't know/Not sure” were not included in this category.

b Women who answered “Don't know/Not sure”.

c The question was stated as “Have birth defects been reported among women infected with Zika during pregnancy?”.

d Among women who knew that birth defects had been reported among pregnant women infected with Zika.

The survey respondents were generally not concerned about Zika affecting their own health, but 33.7% were either very or extremely concerned about Zika affecting their babies' health (Supplemental Fig. 2). After controlling for other sociodemographic characteristics, only race/ethnicity was associated with very or extremely concerned about Zika affecting their own health (Non-Hispanic Whites vs. others, 9.8% vs. 26%, p < 0.001) or their babies' health (31.0% vs. 51.4%, p = 0.003). Almost no pregnant women currently had travel plans to areas with ongoing Zika virus transmissions. Among the 13.8% of participants who previously had plans, most (84.8%) of them cancelled their travel due to Zika.

We performed a sensitivity analysis on survey respondents recruited from Reddit. In total, we received 495 survey respondents from Reddit. After excluding women who refused to provide consent (n = 2), women under 18 years of age (n = 15), women not currently living in the US (n = 34), women not currently pregnant (n = 20), incomplete responses (n = 6), we retained 418 respondents in the sensitivity analysis. The results were similar to those among other respondents who were not recruited from Reddit (Supplemental Table 3).

## 5 Discussion

Almost all women in our sample were highly knowledgeable about Zika virus. Most had heard about Zika and could correctly identify transmission routes and common symptoms. This contrasts with a recent telephone survey (Harvard Opinion Research Program. Harvard T. H. Chan School of Public Health, n.d.) conducted in March of 2016 which found a much lower awareness of Zika virus among the general US population. In their survey, 30% incorrectly identified pneumonia as a possible complication of Zika virus and 31% incorrectly thought that they could get infected by being sneezed or coughed on by someone infected with Zika virus (Harvard Opinion Research Program. Harvard T. H. Chan School of Public Health, n.d.). Less than 80% of their sample followed news on the current outbreak, which is in agreement with another online poll (Ipsos Public Affairs, n.d.) on 1595 adult men and women. The difference in knowledge levels between respondents in our study and participants in the other two studies may be due, in part, to a much higher education level than average among the women we surveyed. In our sample, 39.1% had a master's or doctoral degree, which is much higher than the national average. Another reason for this difference in knowledge levels may have been better access to the internet among our participants, which is a common way to obtain health information (Chiò et al., 2008). Our survey was conducted online and thus was limited to those with internet access. This may also explain why most women in our sample first heard about Zika virus from the internet. In contrast, only 31% used the internet to follow the news about Zika virus in the earlier telephone survey (Harvard Opinion Research Program. Harvard T. H. Chan School of Public Health, n.d.).

Over one third of our respondents had received information about Zika virus from their providers. This is an inordinately quick response from healthcare providers, considering the survey was conducted only a couple months after WHO and CDC issued advisories and travel alerts. The American College of Obstetricians and Gynecologists and Society for Maternal-Fetal Medicine both advise providers to counsel pregnant women about preventive measures for Zika virus (American College of Obstetricians and Gynecologists and Society for Maternal-Fetal Medicine, n.d.). It is important to recognize that time constraints may prevent provider-patient communication regarding issues which do not appear to be of immediate relevance to that office visit (Yi et al., 2015). This may explain why more US providers did not feel the need to discuss a disease in which most cases have been outside the US. Multiple competing demands in the prenatal care setting also distract the attention of providers and pregnant women from conversations about Zika. It is possible that some providers do not feel knowledgeable enough to discuss Zika virus with their patients as it is a new disease to the US. However, the potentially devastating consequences of maternal infection with Zika virus make this discussion an urgent issue. This is especially true now that is known that women may become infected through sexual intercourse and local transmissions of Zika by mosquitos have been reported in Florida. Providers should obtain and provide updated information about Zika to all their pregnant patients to help reduce the risk of a public health crisis related to Zika in the US (Petersen et al., 2016, American College of Obstetricians and Gynecologists and Socie).

This study is limited in its generalizability as most of the participants were recruited from Reddit. However, sociodemographic characteristics between participants from Reddit and other sources were similar and sensitivity analyses also found similar results between those two groups. Well-conducted online surveys have been shown to yield similar results to probability sampling conducted via telephone (Ipsos Public Affairs, 2012). We expected this national sample to be representative of internet-using pregnant women, but homogeneity of the sample indicates that it may not be representative of general pregnant women. Ads which mentioned the word “Zika” may have biased the sample toward women who knew more about the topic. Another limitation is a relatively small sample size. However, little variability was found after the first 30 respondents, so increasing the sample size would probably not have affected our findings.

In this study, most of the respondents were highly-educated white women. We found that 33.4% of the sample reported discussions about Zika with their providers. Further, lower education levels, even among this highly educated sample, was associated with incorrect knowledge about Zika. Therefore, these results reveal a significant gap in communication between providers and their patients about Zika, as only one third women reported receiving information about Zika from health care providers. It is likely more problematic among more socioeconomically-disadvantaged women. This article may be timely: as the mosquito season begins, Florida and other gulf states may possibly see cases from local transmission. Additional efforts are urgently needed to educate providers and pregnant women about how to avoid this disease.

## Conflict of interest disclosures

The authors report no conflict of interest.

## Primary funding sources

Drs. Guo and Fuchs are and Dr. Hirth was supported by a research career development award (K12HD052023: Building Interdisciplinary Research Careers in Women's Health Program-BIRCWH; Berenson, PI) from the Office of Research on Women's Health (ORWH) and the Eunice Kennedy Shriver National Institute of Child Health and Human Development (NICHD) at the National Institutes of Health. Drs. Guo and Fuchs were supported by an institutional training grant (National Research Service Award T32HD055163, Berenson, Principal Investigator) from the Eunice Kennedy Shriver National Institute of Child Health and Human Development of the National Institutes of Health. The content is solely the responsibility of the authors and does not necessarily represent the official views of the National Institutes of Health.

## Role of the funding source

The sponsors had no role in the design and conduct of the study; collection, management, analysis, and interpretation of the data; and preparation, review, or approval of the manuscript, and decision to submit the manuscript for publication.

## Appendix A Supplementary data

Supplementary figures and tables Image 1

Survey questionnaire - English Image 2

Survey questionnaire - Spanish Image 3
