# Supplementary material for: Provider-patient communication about Zika during prenatal visits
Source: Prev Med Rep. 2017 May 18;7:26–9. doi: 10.1016/j.pmedr.2017.05.003 (PMC5447381; doi:10.1016/j.pmedr.2017.05.003)
Supplement: Supplementary file 1 — Supplementary figures and tables [file mmc1.pdf]

Supplemental Figure 1. Numbers of participants from each State in the US (N=492). Southern states are in yellow color, Northeastern states in green color, Midwestern states in blue color, and Western states in red color.

Supplemental Figure 2. Level of concern about Zika affecting their own health or their babies' health among pregnant women (n=469).

Supplemental Figure 1

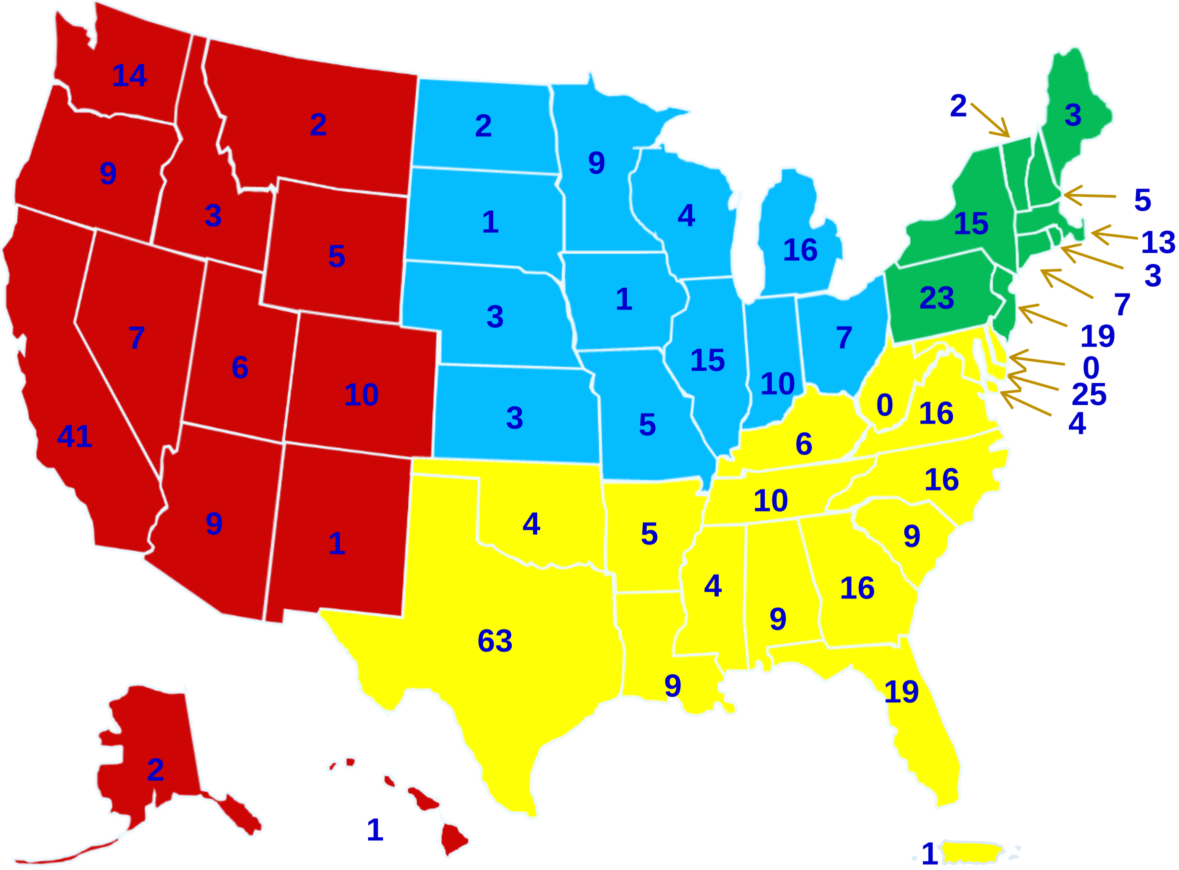

Supplemental Figure 2

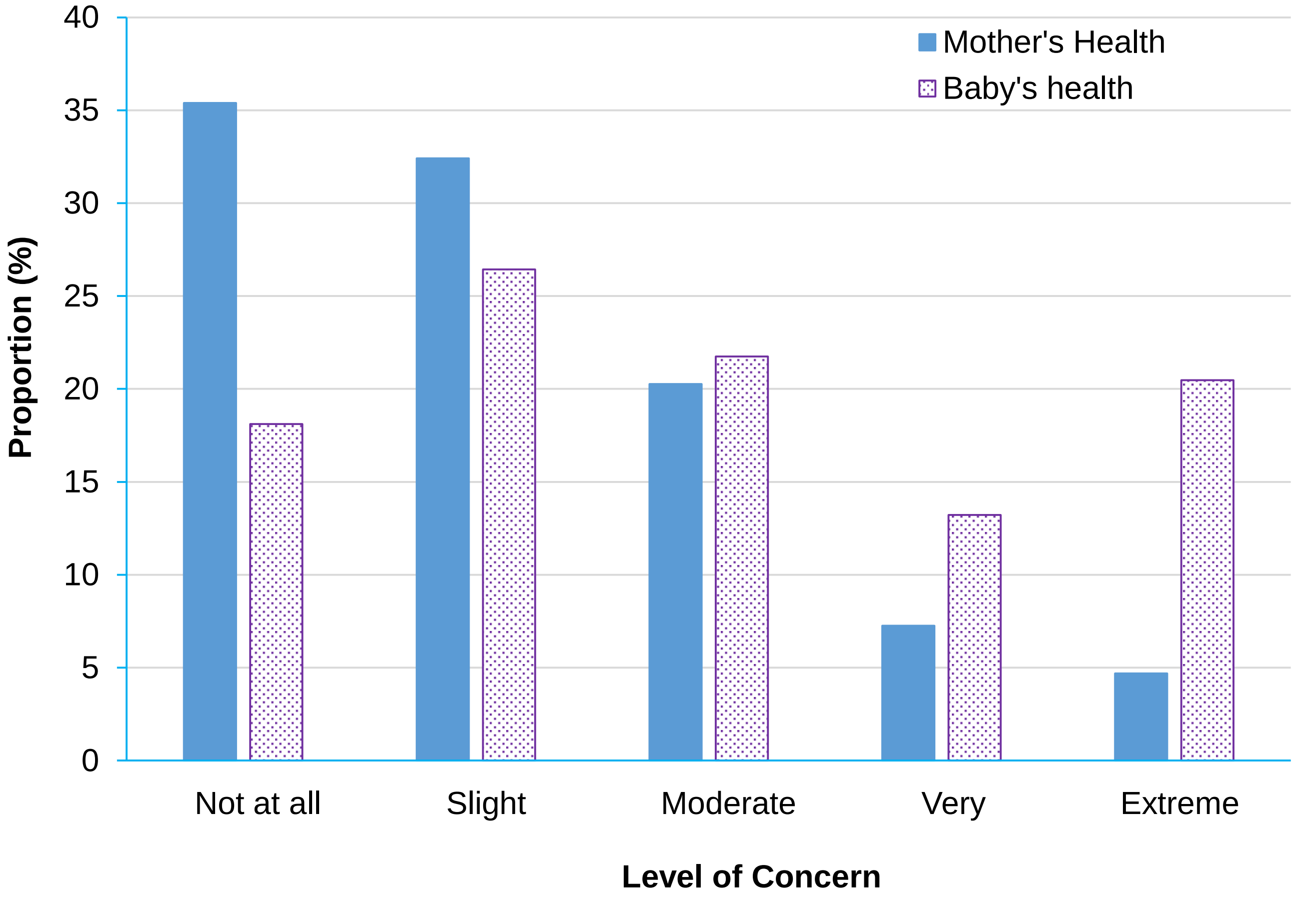

Supplemental Table 1. Sociodemographic characteristics among participants from Reddit and participants from other websites.

	n (%)		
	All	Reddit	Other Sources
**All**	492 (100)	418 (85.0)	74 (15.0)
**Age**			
≤30 years	261 (53.1)	224 (45.5)	37 (7.5)
>30 years	231 (47)	194 (39.4)	37 (7.5)
**Region of residence**			
South	216 (43.9)	178 (36.2)	38 (7.7)
Northeast	90 (18.3)	80 (16.3)	10 (2.0)
Midwest	76 (15.5)	62 (12.6)	14 (2.8)
West	110 (22.4)	98 (19.9)	12 (2.4)
**Country of birth**			
US	458 (93.1)	388 (78.9)	70 (14.2)
Other	34 (6.9)	30 (6.1)	4 (0.8)
**Race/ethnicity**			
Non-Hispanic White	421 (85.6)	365 (74.2)	56 (11.4)
Other	71 (14.4)	53 (10.8)	18 (3.7)
**Education level**			
Master’s or doctoral degree	190 (38.6)	166 (33.7)	24 (4.9)
4-year college degree	189 (38.4)	163 (33.1)	26 (5.3)
No college degree	113 (23)	89 (18.1)	24 (4.9)
**Relationship status**			
Married	426 (86.6)	369 (75.0)	57 (11.6)
Other	66 (13.4)	49 (10.0)	17 (3.5)

Chi-squared and Fisher’s exact tests were used to compare the differences in sociodemographic characteristics between participants from Reddit and participants from other websites. There were no differences in sociodemographic characteristics between participants from Reddit and participants from other sources, except that participants from Reddit had slightly higher proportion of non-Hispanic Whites (87.3% vs. 75.7%, p=0.009) and married women (88.3% vs. 77%, p=0.009) than participants from other sources.

Supplemental Table 2. Health care providers' recommendations regarding travel to areas with Zika outbreaks (n=138).

	Percentage % (95% Confidence Interval)
Avoid travel to areas with Zika outbreaks	94.9 (91.2-98.6)
Use mosquito spray	51.4 (43-59.9)
Wear long-sleeved shirts or long pants	41.3 (33-49.6)
Treat clothing and gear with permethrin	11.6 (6.2-17)
Stay in rooms with air conditioning	32.6 (24.7-40.5)
Sleep under a mosquito bed net	10.1 (5-15.2)

Among pregnant women who reported that their providers discussed risks related to travel to areas with current Zika outbreaks.

Supplemental Table 3. Knowledge about Zika, provider-patient communication about Zika about Zika, and level of concern about Zika affecting their own health or their babies' health among pregnant women from Reddit and other websites.

	Prevalence % (95% CI)	
	Reddit (n=418)	Other Sources (n=74)
**Heard of Zika**	98.8 (97.8-99.8)	91.9 (85.7-98.1)
Heard of Zika from the Internet	74.6 (70.4-78.8)	50.0 (38.1-61.9)
Heard of Zika from TV news	16.0 (12.4-19.5)	39.7 (28.0-51.4)
Aware of CDC travel alerts	90.6 (87.7-93.4)	89.7 (82.5-97.0)
**Provider-patient communication about Zika**	33.3 (28.8-37.9)	33.8 (22.5-45.1)
**Very or extremely concerned about Zika**		
Affecting their own health	11.9 (8.7-15.1)	12.1 (4.2-20.0)
Affecting their babies' health	33.0 (28.4-37.6)	37.9 (26.1-49.6)
**Knowledge about Zika**		
Route of transmission identified incorrectly	4.7 (2.6-6.7)	9.1 (2.1-16.1)
Symptoms identified incorrectly	11.1 (8.0-14.2)	12.1 (4.2-20.0)
Assume cure for Zika infection	1.0 (0.0-2.0)	1.5 (0.0-4.5)
Not know birth defect associated with Zika infection	0.7 (0.0-1.6)	1.5 (0.0-4.5)
Not Microcephaly as the most commonly associated birth defect	0.5 (0.0-1.2)	3.2 (0.0-7.6)
