# Supplementary material for: Provider-patient communication about Zika during prenatal visits
Source: Prev Med Rep. 2017 May 18;7:26–9. doi: 10.1016/j.pmedr.2017.05.003 (PMC5447381; doi:10.1016/j.pmedr.2017.05.003)
Supplement: Supplementary file 2 — Survey questionnaire - English [file mmc2.pdf]

Consent

You are being invited to participate as a subject in the research project entitled "*Knowledge about the Zika virus among pregnant women in the United States*" under the direction of Abbey B. Berenson, MD, PhD, Professor in the Department of Obstetrics & Gynecology, and Director of the Center for Interdisciplinary Research in Women's Health at The University of Texas Medical Branch (UTMB Health).

Purpose of the Study

The purpose of this study is to gather information on knowledge about the Zika virus and travel information among pregnant women in the United States. You are being asked to participate because you are currently pregnant.

Procedures Related Only to the Research

This study will include a brief survey about you and your knowledge about the Zika virus as well as your travel during pregnancy. The survey is expected to take approximately 10-15 minutes to complete. The anticipated number of subjects involved in the study will be 1500. The length of time for your participation in the study is the time it takes to complete the survey.

Risks of Participation

The potential risks from participation in the study are minor emotional discomfort or loss of confidentiality that may arise from participation in the project. If you experience any emotional discomfort while reading or responding to a survey question, you may choose to skip that question or discontinue the survey at any time. There will be no consequences if you choose to skip a question or discontinue the survey. Your information will be kept confidential and stored on a password protected computer in a locked office. You will not be identified by name, email, or any other direct personal identifier in records disclosed outside of UTMB Health. For records disclosed outside of UTMB Health, you will be assigned a unique code number. The key to the code will be kept on a password protected computer in a locked office.

Benefits to the Subject

You will not benefit from your participation in the research project.

Benefits to Society

There are potential benefits to society and the public health. These benefits may include results which inform physicians, health systems, policy-makers, and future research regarding controlling Zika infection and related pregnancy outcomes in the United States.

Costs of Participation

There are no costs to participate in this study.

Additional Information

Participation is voluntary. Refusal to participate will involve no penalty or loss of benefits to which you are otherwise entitled.

Once you submit a response to a question, you will not be able to go back to edit your responses.

Do not use the Forward and Back buttons on your browser to navigate the survey; if you click these buttons, you will exit the survey and not be able to return.

You may contact the principal investigator of this study with any questions or concerns:

Abbey B. Berenson, MD, PhD
Professor, Department of Obstetrics & Gynecology
Director, Center for Interdisciplinary Research in Women's Health
The University of Texas Medical Branch
301 University Blvd.
Galveston, TX 77058-0587
Fax: (409) 747-5129
Email: zika.survey@utmb.edu

If you have any complaints, concerns, input, or questions regarding your rights as a subject participating in this research study or you would like more information, you may contact the Institutional Review Board Office at (409) 266-9475

*** 1. Do you agree to the above terms?**

By selecting "Yes" and clicking the "Next" button, you are indicating that you are at least 18 years old, have read and understood this consent form, and agree to participate in this research study.

- ◯ Yes
- ◯ No

*** 2. Are you a female who is 18 years of age or older?**

- ◯ Yes
- ◯ No

*** 3. Do you currently live in the United States?**

- Yes
- No

*** 4. In what state or U.S. territory do you live?**

*** 5. Are you currently pregnant?**

- Yes
- No

*** 6. How many weeks pregnant are you now?**

*** 7. How old are you (in years)?**

*** 8. What is your country of birth?**

*** 9. How many years have you lived in the United States?**

*** 10. Do you consider yourself to be Hispanic or Latina? This includes:**
Puerto Rican
Cuban/Cuban American
Dominican (Republic)
Mexican
Mexican American
Central or South American
Other Latin American
Other Hispanic/Latina

- ○ Yes
- ○ No

*** 11. What race(s) do you consider yourself to be? Please select 1 or more of these categories.**

- ☐ White
- ☐ Black/African American
- ☐ Native American
- ☐ Alaska Native
- ☐ Native Hawaiian
- ☐ Guamanian or Chamorro
- ☐ Samoan
- ☐ Other Pacific Islander
- ☐ Asian Indian
- ☐ Chinese
- ☐ Filipino
- ☐ Japanese
- ☐ Korean
- ☐ Vietnamese
- ☐ Other Asian
- ☐ Some other race (please specify)

*** 12. What is the highest level of school you have completed?**

- Never attended/kindergarten only
- Primary school (1st grade to 8th grade)
- Some high school, but no diploma
- High school diploma (or GED)
- Some college, but no degree
- 2-year college degree
- 4-year college degree
- Master’s or Doctoral degree

*** 13. Which of the following best describes your current relationship status?**

- Married
- Widowed
- Divorced
- Separated
- Living with partner
- Single, never married

*** 14. Have you ever heard of the Zika virus?**

- Yes
- No

*** 15. Where did you first hear about the Zika virus?**

- ◯ TV news
- ◯ Internet
- ◯ Health care provider or clinic
- ◯ Newspaper
- ◯ Friend or relative
- ◯ Other source (please specify)

*** 16. Are you aware of the Centers for Disease Control and Prevention (CDC) recommendations for pregnant women regarding travel to areas with Zika outbreaks?**

- ◯ Yes
- ◯ No
- ◯ Don't know / Not sure

*** 17. Has your healthcare provider talked with you about the Zika virus?**

- ◯ Yes
- ◯ No

*** 18. Has your health care provider talked with you about risks to pregnant women related to travel to areas with Zika outbreaks?**

- ◯ Yes
- ◯ No

19. What did your health care provider tell you about travel to areas with Zika outbreaks? Please select all that apply.

- [ ] Avoid travel to these areas
- [ ] Use mosquito spray
- [ ] Wear long-sleeved shirts or long pants
- [ ] Treat clothing and gear with permethrin
- [ ] Stay in places with air conditioning or with window and door screens to keep mosquitoes outside
- [ ] Sleep under a mosquito bed net
- [ ] Other (please specify)

Now we would like to ask you some questions about the Zika virus. If you do not know the answer, check the box marked "Don't know / Not sure".

*** 20. Can people get infected with the Zika virus from:**

	Yes	No	Don't know / Not sure
Sexual contact with an infected human?	○	○	○
Through sharing air with an infected person, especially if they are coughing?	○	○	○
The bite of an infected mosquito?	○	○	○

*** 21. In which of the following countries or regions is the Zika virus currently spreading locally by mosquito bites? Please choose all that apply.**

- [ ] Continental U.S.
- [ ] Mexico
- [ ] Central America
- [ ] Brazil
- [ ] Alaska
- [ ] Colombia
- [ ] Puerto Rico
- [ ] Caribbean islands

*** 22. Please indicate whether or not each of the following symptoms can occur as a result of infection with the Zika virus:**

	Yes	No	Don't know / Not sure
Fever	○	○	○
Rash	○	○	○
Joint pain	○	○	○
Conjunctivitis (red eyes)	○	○	○
Muscle pain	○	○	○
Headache	○	○	○

*** 23. Is there a cure for Zika infection?**

○ Yes

○ No

○ Don't know / Not sure

*** 24. Have birth defects been reported among women infected with Zika during pregnancy?**

- ◯ Yes
- ◯ No
- ◯ Don’t know / Not sure

25. Which birth defect has been most commonly reported among women infected with Zika during pregnancy?

- ◯ Heart problems
- ◯ Microcephaly (small head)
- ◯ Short gut
- ◯ None of the above
- ◯ Don't know

Next, we would like to ask you some questions about your travel.

*** 26. How many times did you travel to any of the following countries/regions in the<u>past 12 months</u>?**

	0	1	2	3	4 or more times
Mexico	◯	◯	◯	◯	◯
Central America	◯	◯	◯	◯	◯
Brazil	◯	◯	◯	◯	◯
Colombia	◯	◯	◯	◯	◯
Puerto Rico	◯	◯	◯	◯	◯
Caribbean islands	◯	◯	◯	◯	◯

*** 27. When you traveled to one of these areas last year, how often did you use each of the following to protect yourself against mosquito bites?**
If used, but not as a protection against mosquito bites, check the last box.

	Used often	Used occasionally	Didn't use at all	Used, but not because of mosquitoes
Mosquito repellent spray	○	○	○	○
Long-sleeved shirts, long pants	○	○	○	○
Treat clothing and gear with permethrin	○	○	○	○
Stay in places with air conditioning or with window and door screens to keep mosquitoes outside	○	○	○	○
Sleep under a mosquito bed net	○	○	○	○

*** 28. Did you PREVIOUSLY have travel plans to any of the following areas during this pregnancy, but changed your plans because you were concerned about Zika?**
Mexico, Central America, Brazil, Colombia, Puerto Rico, Caribbean islands

○ Yes

○ No

○ Never had plans to travel to any of those areas during this pregnancy

*** 29. Do you CURRENTLY have plans to travel to any of the following areas during this pregnancy?**
Mexico, Central America, Brazil, Colombia, Puerto Rico, Caribbean islands

○ Yes

○ No

○ Don’t know / not sure

*** 30. How concerned are you about Zika affecting YOUR health?**

- ◯ Extremely concerned
- ◯ Very concerned
- ◯ Moderately concerned
- ◯ Slightly concerned
- ◯ Not at all concerned

*** 31. How concerned are you about Zika affecting the health of YOUR BABY?**

- ◯ Extremely concerned
- ◯ Very concerned
- ◯ Moderately concerned
- ◯ Slightly concerned
- ◯ Not at all concerned

Next, we would like to ask you about mosquito bites.

*** 32. When you were outdoors during the mosquito season in the past year in the United States, how often did you use each of the following to protect yourself against mosquito bites?**
If used but not as a protection against mosquito bites, check the last box.

	Used often	Used occasionally	Didn't use at all	Used, but not because of mosquitoes
Mosquito repellent spray	◯	◯	◯	◯
Long-sleeved shirts, long pants	◯	◯	◯	◯
Treated clothing and gear with permethrin	◯	◯	◯	◯

*** 33. Please indicate your level of agreement with the following statements.**

	Strongly agree	Agree	Neither agree nor disagree	Disagree	Strongly disagree	Not applicable
Mosquito bites do not bother me.	○	○	○	○	○	○
It is important to avoid getting mosquito bites.	○	○	○	○	○	○
It's hard to get the kind of insect repellent that I prefer.	○	○	○	○	○	○
Insect repellent is easy to find.	○	○	○	○	○	○
Insect repellent is too expensive.	○	○	○	○	○	○
Proper, fitted screens are too hard to get.	○	○	○	○	○	○
Proper, fitted screens are too expensive.	○	○	○	○	○	○
There are a lot of mosquitoes around my home.	○	○	○	○	○	○
It is important for people to reduce the number of mosquitoes around their homes.	○	○	○	○	○	○

*** 34. Would you like to take future surveys about Zika?**

○ Yes

○ No

35. Please enter the best email address where you can be contacted for future survey opportunities.

If you do not wish to disclose your email address, you can exit the survey.
