# Supplementary material for: Provider-patient communication about Zika during prenatal visits
Source: Prev Med Rep. 2017 May 18;7:26–9. doi: 10.1016/j.pmedr.2017.05.003 (PMC5447381; doi:10.1016/j.pmedr.2017.05.003)
Supplement: Supplementary file 3 — Survey questionnaire - Spanish [file mmc3.pdf]

Consentimiento

Usted está invitada a participar como sujeto en el proyecto de investigación titulado *“Conocimiento acerca del virus Zika entre mujeres embarazadas en los Estados Unidos”* bajo la dirección de Abbey B. Berenson , MD, PhD, profesora en el Departamento de Obstetricia y Ginecología y Directora del Centro de investigaciones interdisciplinarias en salud de las mujeres en La Universidad de Texas Medical Branch (UTMB Health).

El propósito del estudio

El propósito de este estudio es reunir información sobre el conocimiento del virus Zika y viajes entre las mujeres embarazadas en los Estados Unidos. A usted se le pide participar porque está actualmente embarazada.

Los procedimientos relacionados solamente por la investigación

Este estudio incluirá una encuesta breve sobre usted y su conocimiento sobre el virus Zika, así como sus viajes durante el embarazo. La encuesta debe tomar aproximadamente 10-15 minutos para completar. El número previsto de sujetos involucrados en el estudio será 1500. La duración que le tome su participación en el estudio es el tiempo que toma para completar la encuesta.

Los riesgos de la participación

Los riesgos potenciales de la participación en el estudio son un malestar emocional leve o pérdida de la confidencialidad que surja de la participación en el proyecto. Si experimenta cualquier malestar emocional mientras lee o respondiendo a una pregunta de la encuesta, usted puede omitir esta pregunta o descontinuar la encuesta en cualquier momento. No habrá ninguna consecuencia si opta por omitir una pregunta o discontinuar la encuesta. Su información se mantendrá confidencial y la almacenados en una computadora protegida por contraseña en una oficina cerrada. Usted no será identificado por nombre, correo electrónico o cualquier otro identificador personal directo en registros divulgados fuera de UTMB Health. Para los registros divulgados fuera de UTMB Health, se le asignará a un número de código único. La clave para el código se mantendrá en una computadora protegida por contraseña en una oficina cerrada.

Los beneficios para el sujeto

Usted no se beneficiará de su participación en el proyecto de

Los beneficios para la sociedad

Existen beneficios potenciales para la sociedad y para la salud pública. Estos beneficios pueden incluir resultados que informan a los médicos, a los sistemas de salud, a los responsables políticos y la investigación futura en cuanto al control de la infección de Zika  y los resultados del embarazo relacionados en los Estados Unidos

Los costos de la participación

No hay ningún costo para participar en este estudio.

Información adicional

La participación es voluntaria. El rechazo a participar no implicará penalización ni pérdida de beneficios a los cuales usted tiene derecho.

Una vez que someta una respuesta a una pregunta, no podrá volver a editar sus respuestas.

**No utilice los botones del navegador para navegar hacia a adelante y atrás en la encuesta; Si hace clic en estos botones, puede salirse completamente de la encuesta y no será posible regresarse.**

Usted puede ponerse en contacto con el investigador principal de este estudio con sus preguntas o dudas:

Abbey B. Berenson, MD, PhD
Profesora, del Departamento de Obstetricia & Ginecología
Directora, del Centro para Investigaciones interdisciplinarias en salud de la mujer
La Universidad de Texas Medical Branch
301 University Blvd.
Galveston, TX 77058-0587
Fax: (409) 747-5129
Email: zika.survey@utmb.edu

Si usted tiene quejas, preocupaciones, aportaciones o preguntas con respecto a sus derechos como un sujeto en participar en este estudio de investigación o le gustaría obtener más información, puede comunicarse con la oficina de Junta de revisión institucional en (409) 266-9475.

**1. ¿Está de acuerdo con los términos de arriba?**

**Mediante seleccionado "Si" y haciendo clic en el botón "Siguiente", usted indica que tiene al menos 18 años de edad, y ha leído y entendido esta forma de consentimiento y está de acuerdo en participar en este estudio de investigación.**

- ◯ Si
- ◯ No

**2. ¿Eres una mujer de 18 años de edad o más?**

- ◯ Si
- ◯ No

**3. ¿Actualmente vive usted en los Estados Unidos?**

- ◯ Si
- ◯ No

**4. ¿En qué estado o territorio de Estados Unidos vive usted?**

**5. ¿Esta usted embarazada actualmente?**

- ◯ Si
- ◯ No

**6. ¿Cuántas semanas de embarazo tiene usted ahora?**

**7. ¿Qué edad tiene usted? (en años)**

**8. ¿Cuál es su país de nacimiento?**

**9. ¿Cuántos años ha vivido en los Estados Unidos?**

**10. ¿Se considera usted como hispana o latina? Esto incluye:**
**Puerto Rico/Puertorriqueña**
**Cuba/Cubana Americana**
**Republica Dominicana**
**Mexicana**
**Mexicana Americana**
**Centro/Sudamericana**
**América Latina otra**
**Hispania/Latina otra**

- ◯ Si
- ◯ No

**11. ¿Qué raza(s), se considera ser? Por favor seleccione 1 o más de estas categorías.**

- ☐ Blanca
- ☐ Negro/Afro-American
- ☐ Nativo americano
- ☐ Alaska nativo
- ☐ Hawaiano Nativo
- ☐ Guameños o Chamorro
- ☐ Samoano
- ☐ Otros isleños del pacifico
- ☐ India asiática
- ☐ Chino
- ☐ Filipino
- ☐ Japonés
- ☐ Coreano
- ☐ Vietnamita
- ☐ Otros de Asia
- ☐ Otra raza (por favor especifique)

**12. ¿Cuál es el nivel más alto de escuela que ha terminado?**

- ◯ Nunca asistió/solamente el kínder
- ◯ Escuela primaria (1er grado a 8vo grado)
- ◯ Algo de preparatoria, pero sin diploma
- ◯ Diploma de secundaria/preparatoria o (GED)
- ◯ Algo de Universidad, pero sin tutulo
- ◯ 2 años de título universitario
- ◯ 4 años de título universitario
- ◯ Título de maestría o doctorado

**13. ¿Cuál de los siguientes describe mejor su relación/estado civil?**

- ◯ Casada
- ◯ Viuda
- ◯ Divorciada
- ◯ Separada
- ◯ Viendo juntos
- ◯ Soltera, nunca casada

**14. ¿Ha escuchado alguna vez hablar sobre el virus Zika?**

- ◯ Si
- ◯ No

**15. ¿Cuándo por primera vez escucho sobre el virus Zika?**

- ◯ Noticias de TV
- ◯ Internet
- ◯ Médico o clínica
- ◯ Periódico
- ◯ Amigo o pariente
- ◯ Otra fuente (por favor, especifique)

**16. ¿Está usted consciente que los centros para el Control de enfermedades y prevención de enfermedades (CDC) a dado recomendaciones para las mujeres embarazadas con respecto a viajar a zonas con brotes del virus Zika?**

- ◯ Si
- ◯ No
- ◯ No sé / no está segura

**17. ¿Ha hablado su médico/proveedor de salud con usted sobre el virus Zika?**

- ◯ Si
- ◯ No

**18. ¿Ha hablado su médico/proveedor de salud con usted sobre los riesgos para las mujeres embarazadas que viajan a zonas con brotes del virus Zika?**

- ◯ Si
- ◯ No

**19. ¿Qué le dijo su médico/proveedor de salud sobre los viajes a zonas con brotes de Zika? Por favor seleccione todas las que apliquen.**

- [ ] Evitar viajar a estas áreas
- [ ] Utilice repelente de mosquito
- [ ] Use camisas de manga larga o pantalones largos
- [ ] Trate la ropa y el equipo con permetrina
- [ ] Alojamiento que tenga aire acondicionado o malla de tela metálica en las puertas y ventanas para mantener los mosquitos afuera
- [ ] Duerma debajo de un mosquitero
- [ ] Otros (especifique)

**Ahora nos gustaría hacerle algunas preguntas sobre el virus Zika. Si no sabe la respuesta, marque la casilla marcada "no se / no está segura".**

**20. Puede la gente infectarse con el virus Zika por:**

| | Si | No | No sé/ no está segura |
|---|---|---|---|
| ¿Teniendo contacto sexual con un ser humano infectado? | ○ | ○ | ○ |
| ¿A través de compartir aire con una persona infectada, especialmente si están tosiendo? | ○ | ○ | ○ |
| ¿Por medio de la picadura de un mosquito infectado? | ○ | ○ | ○ |

**21. ¿En cuál de los siguientes países o regiones está el virus Zika actualmente propagándose localmente por picaduras de mosquito? Por favor seleccione todos los que apliquen.**

- ☐ Continental de EE.UU.
- ☐ México
- ☐ Centroamérica
- ☐ Brasil
- ☐ Alaska
- ☐ Colombia
- ☐ Puerto Rico
- ☐ Las islas del Caribe

**22. Por favor indique si los siguientes síntomas pueden ocurir como resultado de una infección del virus Zika, por favor indique "si" o "no" a cada uno de los siguientes síntomas:**

| | Si | No | No sé/ no está segura |
|---|---|---|---|
| Fiebre | ○ | ○ | ○ |
| Salpullido | ○ | ○ | ○ |
| Dolor de articulacin | ○ | ○ | ○ |
| Conjuntivitis (ojos rojos) | ○ | ○ | ○ |
| Dolor muscular | ○ | ○ | ○ |
| Dolor de cabeza | ○ | ○ | ○ |

**23. ¿Existe una cura para la infección de Zika?**

- ○ Si
- ○ No
- ○ No sé/ no está segura

**24. ¿Se han reportado defectos de nacimiento en las mujeres infectadas con Zika durante el embarazo?**

- ◯ Si
- ◯ No
- ◯ No sé/ no está segura

**25. ¿Qué defectos de nacimiento han sido reportados con mayor frecuencia entre las mujeres infectadas con Zika durante el embarazo?**

- ◯ Problemas del corazón
- ◯ Microcefalia (cabeza pequeña)
- ◯ intestino corto
- ◯ Ninguna de las anteriores
- ◯ No se

**A continuación, nos gustaría hacerle algunas preguntas sobre su viaje.**

**26. ¿Cuántas veces tuvo que viajar a cualquiera de los siguientes países/regiones en los últimos 12 meses?**

| | 0 | 1 | 2 | 3 | 4 o mas |
|---|---|---|---|---|---|
| México | ◯ | ◯ | ◯ | ◯ | ◯ |
| Centroamérica | ◯ | ◯ | ◯ | ◯ | ◯ |
| Brasil | ◯ | ◯ | ◯ | ◯ | ◯ |
| Colombia | ◯ | ◯ | ◯ | ◯ | ◯ |
| Puerto Rico | ◯ | ◯ | ◯ | ◯ | ◯ |
| Las islas del Caribe | ◯ | ◯ | ◯ | ◯ | ◯ |

**27. ¿Cuándo usted viajó a una de estas áreas el año pasado, con qué frecuencia uso cada una de las siguientes opciones para protégerse contra las picaduras de mosquitos?**
**Si utiliza estos métodos, pero no como una protección contra las picaduras de mosquito, por favor marque la última casilla.**

| | Utiliza a menudo | Utiliza de vez en cuando | No usar en absoluto | Utiliza, pero no por los mosquitos |
|---|---|---|---|---|
| Repelente/espray de insectos | ◯ | ◯ | ◯ | ◯ |
| Camisas de manga larga y pantalones largos | ◯ | ◯ | ◯ | ◯ |
| Usa permetrina para tratar la ropa y el equipo | ◯ | ◯ | ◯ | ◯ |
| Alojamiento que tenga aire acondicionado o malla de tela metálica en las puertas y ventanas para mantener los mosquitos afuera | ◯ | ◯ | ◯ | ◯ |
| Duerma debajo de un mosquitero | ◯ | ◯ | ◯ | ◯ |

**28. ¿Tuvo anteriormente planes para viajar a cualquiera de las siguientes áreas durante este embarazo, pero cambió sus planes porque estaba preocupado por el virus Zika?**
**México, Centroamérica, Brasil, Colombia, Puerto Rico, Las islas del Caribe**

◯ Si

◯ No

◯ No tuve planes de viajar a cualquiera de esas áreas durante este embarazo

**29. ¿Tiene usted planes para viajar a cualquiera de las siguientes áreas durante este embarazo?**
**México, Centroamérica, Brasil, Colombia, Puerto Rico, Las islas del Caribe**

◯ Si

◯ No

◯ No sé/ no está segura

**30. ¿Cuánto le preocupa que el virus Zika afectesu salud?**

- Extremadamente preocupada
- Muy preocupada
- Moderadamente preocupada
- un poco preocupada
- No, en absoluto preocupada

**31. ¿Cuánto le preocupa que el virus Zika afecte la salud desu bebé?**

- Extremadamente preocupada
- Muy preocupada
- Moderadamente preocupada
- un poco preocupada
- No, en absoluto preocupada

**A continuación, nos gustaría preguntarle acerca de las picaduras de mosquitos.**

**32. Cuando estuvo al aire libre durante la temporada de mosquitos en el último año en los Estados Unidos, ¿con qué frecuencia uso cada una de las siguientes opciones para protegerse contra las picaduras de mosquitos?**
**Si utiliza estos métodos, pero no como una protección contra las picaduras de mosquito, por favor marque la última casilla.**

| | Utiliza a menudo | Utiliza de vez en cuando | No usar en absoluto | Utiliza, pero no por los mosquitos |
|---|---|---|---|---|
| Repelente/espray de insectos | ○ | ○ | ○ | ○ |
| Camisa de manga larga y pantalones largos | ○ | ○ | ○ | ○ |
| Usa permetrina para tratar la ropa y el equipo | ○ | ○ | ○ | ○ |

### 33. Por favor indique su nivel de acuerdo con las siguientes declaraciones

| | Muy de acuerdo | De acuerdo | Ni de acuerdo ni en desacuerdo | Desacuerdo | Muy en desacuerdo | No aplica |
|---|---|---|---|---|---|---|
| Las picaduras de mosquitos no me molestan. | ◯ | ◯ | ◯ | ◯ | ◯ | ◯ |
| Es importante evitar las picaduras de mosquitos. | ◯ | ◯ | ◯ | ◯ | ◯ | ◯ |
| Es difícil obtener el tipo de repelente de insectos que prefiero. | ◯ | ◯ | ◯ | ◯ | ◯ | ◯ |
| El repelente de insectos es fácil de encontrar. | ◯ | ◯ | ◯ | ◯ | ◯ | ◯ |
| El repelante de insectos es demasiado caro. | ◯ | ◯ | ◯ | ◯ | ◯ | ◯ |
| Malla de tela metálica/mosquiteros adecuada son difícil de obtener. | ◯ | ◯ | ◯ | ◯ | ◯ | ◯ |
| Malla de tela metálica/mosquiteros son muy caros. | ◯ | ◯ | ◯ | ◯ | ◯ | ◯ |
| Hay una gran cantidad de mosquitos alrededor de mi casa | ◯ | ◯ | ◯ | ◯ | ◯ | ◯ |
| Es importante para las personas a reducir el número de mosquitos alrededor de sus hogares. | ◯ | ◯ | ◯ | ◯ | ◯ | ◯ |

### 34. ¿Le gustaría participar en futuras encuestas sobre Zika?

◯ Si

◯ No

**35. Por favor, indique su mejor correo electrónico donde se le pueda contactar para futuras oportunidades de estudio.**

**Si no desea revelar su dirección de correo electrónico, puede finalizar la encuesta.**
